# Evidence for Enhanced Interoceptive Accuracy in Professional Musicians

**DOI:** 10.3389/fnbeh.2015.00349

**Published:** 2015-12-17

**Authors:** Katharina L. Schirmer-Mokwa, Pouyan R. Fard, Anna M. Zamorano, Sebastian Finkel, Niels Birbaumer, Boris A. Kleber

**Affiliations:** ^1^Institute for Medical Psychology and Behavioural Neurobiology, University of TübingenTübingen, Germany; ^2^School of Psychology, Technical University of DresdenDresden, Germany; ^3^Research Institute on Health Sciences, University of Balearic IslandsPalma de Mallorca, Spain; ^4^Ospedale San Camillo, Istituto di Ricovero e Cura a Carattere ScientificoVenice, Italy; ^5^International Laboratory for Brain, Music and Sound ResearchMontreal, QC, Canada

**Keywords:** interoception, musicians, insula, multisensory integration, heartbeat perception

## Abstract

Interoception is defined as the perceptual activity involved in the processing of internal bodily signals. While the ability of internal perception is considered a relatively stable trait, recent data suggest that learning to integrate multisensory information can modulate it. Making music is a uniquely rich multisensory experience that has shown to alter motor, sensory, and multimodal representations in the brain of musicians. We hypothesize that musical training also heightens interoceptive accuracy comparable to other perceptual modalities. Thirteen professional singers, twelve string players, and thirteen matched non-musicians were examined using a well-established heartbeat discrimination paradigm complemented by self-reported dispositional traits. Results revealed that both groups of musicians displayed higher interoceptive accuracy than non-musicians, whereas no differences were found between singers and string-players. Regression analyses showed that accumulated musical practice explained about 49% variation in heartbeat perception accuracy in singers but not in string-players. Psychometric data yielded a number of psychologically plausible inter-correlations in musicians related to performance anxiety. However, dispositional traits were not a confounding factor on heartbeat discrimination accuracy. Together, these data provide first evidence indicating that professional musicians show enhanced interoceptive accuracy compared to non-musicians. We argue that musical training largely accounted for this effect.

## Introduction

Interoception refers to the perception of internal bodily sensations from all organs (i.e., viscera as well as the muscles and joints), which provides the individual with information on the body's ongoing physiological condition and contributes toward guiding behavior (Ádám and Pennebaker, 1998; Craig, 2002, 2003). These somatic sensations, which also affect cognition and emotion, may occur together with specific context or in the absence of external stimuli (Michael et al., 2015). Research has indicated, however, that both external and internal sensations are highly interrelated and require multisensory integration to jointly contribute to body awareness (Craig, 2009; Aspell et al., 2013; Simmons et al., 2013; Suzuki et al., 2013). Although everyday life events mostly depend on multisensory integration (Paraskevopoulos et al., 2012), musicians with their extensive sensorimotor training have developed a unique ability to simultaneously integrate feedback from different sensory modalities (Altenmüller, 2008). This is reflected by a strengthening of associative connections between motor, sensory, and multimodal integration areas in the brain (for reviews, see Herholz and Zatorre, 2012; Schlaug, 2015). Despite numerous studies reporting neuroplastic and behavioral effects of musical training, it is not clear from these data whether enhanced multisensory integration might also boost interoceptive accuracy or if the specific kind of musical training could affect interoceptive accuracy differentially.

Interoceptive accuracy has been considered a relatively robust and independent personality trait (Schandry, 1981; Ainley et al., 2013), which may be the reason why explicit entrainment of internally focused attention has failed to enhance interoception (Khalsa et al., 2008; Daubenmier et al., 2013). Recent reports, however, suggest that heightened attention to the body can be achieved by integrating self-relevant external (e.g., visual and/or acoustic) cues (Ainley et al., 2013; Suzuki et al., 2013). At the neural level, multimodal integration has been associated with activity of the right anterior insula (AI), which has been described as the neuroanatomical correlate of awareness of bodily feelings (Critchley, 2004; Craig, 2009; Seth et al., 2011; Critchley and Harrison, 2013). Neuroimaging studies with trained musicians reported a significantly larger causal interaction output from the AI compared to non-musicians (Luo et al., 2012). Moreover, it has been shown that the AI responded differently in trained and untrained singers during singing with anesthetized vocal fold mucosa (Kleber et al., 2013). These data indicate that the AI gates feedback and feedforward motor-control mechanisms as a function of musical training, which is in line with the involvement of the insula in the acquisition of action-perception links during music learning (Mutschler et al., 2007).

Other sources of visceral signals that can alter the perception of bodily states are painful stimulation and emotional experiences (Critchley et al., 2001; Craig, 2002, 2009; Singer et al., 2004; Baumgartner et al., 2009), congruent with the “James–Lange” theory of emotion (James, 1884) and Damasio's somatic marker hypothesis (Damasio, 1996; Craig, 2004). Pain processing is associated with visceral feelings that activate insular cortex (Baliki et al., 2006; Craig, 2010; Tsay et al., 2015). Interestingly, up to 86% of professional musicians are affected by musculoskeletal pain and pain-related symptoms (Steinmetz et al., 2015), mostly due to repetitive-strain injuries as a consequence of extensive practice with the instrument (Brandfonbrener, 2003; Steinmetz and Jull, 2013). In addition, professional musical training may also lead to enhanced pain sensitivity in healthy musicians in the absence of concurrent pain syndromes (Zamorano et al., 2014).

On the other hand, individuals who are more attuned to their bodily responses may also experience their emotions with heightened arousal and valence (Wiens et al., 2000; Garfinkel and Critchley, 2013). In the same vain, excessive emotionality and mood disorders have been associated with somatic hypervigilance and increased insular activity in both clinical and non-clinical populations, suggesting that interoception plays a critically important role in the appraisal of emotions (Domschke et al., 2010; Dunn et al., 2010; Mallorqui-Bague et al., 2014). Music, a potent trigger of discrete emotions, engages the insula via the autonomic arousal of bodily reactions (Koelsch, 2014). Musicians, however, may not only experience emotions based on the affective content of music but also due to increased arousal levels as a result of public performance situations. Musical performance anxiety (MPA) is a widespread phenomenon that has been associated with a psychological vulnerability to experience negative emotions and a shift toward a self-evaluative focus in the context of public performance (Barlow, 2000; Kenny et al., 2004). Despite some overlap, however, debilitating MPA can be distinguished from general trait or social anxiety (Fehm and Schmidt, 2006; Simoens et al., 2015). Moreover, it may be lower in extraverted individuals (Stemmler and Wacker, 2010; Thomas and Nettelbeck, 2014). The assessment of interoceptive accuracy should therefore also consider potential differences in dispositional traits in the sample under investigation.

In the current study, we examined interoceptive accuracy with a well-known heartbeat-discrimination task (Brener and Kluvitse, 1988). In this task, participants are first presented with a series of acoustic stimuli triggered by their ECG R-peak and subsequently asked to report whether the perceived acoustic feedback was synchronous with their internally perceived heartbeat or not (Critchley et al., 2004). As this procedure requires simultaneous multimodal integration of internal and external information, it was preferred over heartbeat tracking tasks in which the focus is laid on internal monitoring (i.e., counting) of heartbeats without integrating external information (see Schandry, 1981; Garfinkel et al., 2015). We compared musically untrained individuals against two homogeneous samples of professional musicians with well-distinguishable sensorimotor skills (i.e., vocalists and string players) to assess the incremental validity of subject-specific musical training on interoceptive accuracy. Furthermore, psychometric data were collected to evaluate interactions between interoceptive accuracy and dispositional traits.

We predicted that professional musicians would be significantly more accurate in heartbeat-detection than non-musicians as a consequence of musical training. We expected this effect to be independent from MPA, emotional, and dispositional traits, which were anticipated to be within normal limits across groups. Furthermore, we speculated that singers could be more aware of their heartbeats than string players, as singers use a more body-core centered and visceral nature of music production, involving organs and muscles of vital importance (Wild, 2004; Kleber et al., 2010).

## Methods

### Participants

Two groups of professional musicians and one group of non-musicians with a total of 38 right-handed individuals participated in this study. Exclusion criteria were reported history of neurological or psychiatric diseases. Selection criteria for musicians were based on (a) the visceral involvement and (b) the distinctness of sensorimotor skills for music production. Correspondingly, we selected two homogenous groups of professionally trained classical singers (*n* = 13, mean age: 27 year, SD: 3.6; 46% female) and professionally trained string players (*n* = 12, mean age: 25.3 year, SD: 2.6; 50% female). Singers consisted of three sopranos, four mezzo-sopranos, three tenors, one baritone, and two basses. Strings consisted of five violin players, three viola players, and four violoncello players. The control group consisted of age and sex matched non-musicians (*n* = 13, mean age: 27.7 year, SD: 3.5; 54% female), who never received any formal or informal musical training. All subjects gave written informed consent before participation. The study was conducted under a protocol approved by the research ethics board of the University of Tübingen in agreement with the Declaration of Helsinki.

### Musical experience

Table 1 provides information about the age of commencement with formal training and the entry age to a music conservatory respectively, the total years of training, and the hours of current weekly practice. To estimate the total amount of accumulated musical experience, we multiplied the years of formal training with the hours of weekly practice and the weeks per year. Based on this rough approximation (i.e., years of training^*^weekly singing practice ^*^52), singers trained on average about 6440 h (range: 2.184–13.104, *SD* = 3673) and string-players on average about 19.367 h (range: 9.282–36.400, *SD* = 9419).

**Table 1:** **Means and standard deviations (S.D.) for demographic characteristics, heartbeat-detection accuracy, musical experience, and psychometric test scores**.

	**Musicians**		**Non-musicians**
	**Singers (*n* = 13)**	**String (*n* = 12)**	**(*n* = 13)**
Age	27 (3.6)	25.3 (2.6)	27.7 (3.4)
Sex (% female)	46	50	54
**INTEROCEPTIVE ACCURACY**			
Heartbeat detection (% corr.)	64.3 (18.7)	62.5 (18.9)	48.6 (8.9)
**MUSICAL TRAINING**			
Age training commenced	17.3 (2.8)	6.8 (1.0)	n.a.
Age at music conservatory	20.9 (2.8)	18.1 (2.2)	n.a.
Weekly practice (h)	12.4 (3.9)	19.8 (8.1)	n.a.
Years of training	9.7 (3.9)	18.5 (2.7)	n.a.
Estimated acc. training (h)	6440 (3673)	19367 (9419)	
**ANXIETY AND DEPRESSION**			
MPA solo	46.3 (9.9)	59.7 (12.7)	n.a.
MPA ensemble	36.2 (5.0)	44.0 (13.2)	n.a.
STAI-T	37.6 (12.2)	41.8 (9.2)	40.5 (9.7)
CESD	10.7 (6.2)	12.8 (6.9)	11.6 (6.0)
**SELF-ATTENTION**			
SCS private	54.2 (10.7)	52.8 (5.6)	53.5 (9.2)
SCS public	53.5 (13.2)	56.3 (7.9)	53.5 (6.0)
PVAQ	36.5 (11.1)	28.3 (14.9)	29.1 (18.0)
**PERSONALITY**			
Extraversion	27.8 (8.0)	28.0 (5.6)	30.0 (5.6)

*MPA, musical performance anxiety; STAI-T, trait anxiety questionnaire; CESD, Center for Epidemiologic Studies Depression Scale; SCS, self-consciousness scale; PVAQ, pain vigilance and awareness questionnaire. Citations of corresponding German versions are indicated in the text*.

### Electrocardiographic (ECG) recordings

ECG data were recorded from each participant using a portable Nexus 10 device (Mind Media BV., http://www.mindmedia.info/) connected via Bluetooth to a laptop computer. Positive, negative, and ground ECG electrodes were attached to the participant's chest with a lead II placement, which produces the largest positive R-wave. R-peak detection was performed online using MATLAB signal processing toolbox (Mathworks Inc., http://www.mathworks.com/). The ECG data were recorded at a 1000 Hz sampling-rate into a buffer and streamed, every 100 ms, through the processing pipeline. Upon arrival of new data, a second-order band-pass Butterworth filter was applied. A simple automated method with fixed threshold was used for detecting the peaks of the ECG R-waves. The threshold was set by running a 1-min baseline recording and was updated every 7 trials to prevent drift-effects in the ECG waves. Heartbeats were presented with a pure tone of 100 ms duration at a frequency of 800 Hz. The buffering and processing delays were computed in order to enable the program to accurately impose the desired amount of time delay between the R-peak and the played tone.

### Heartbeat discrimination task

Interoceptive accuracy was measured using a popular heartbeat discrimination paradigm (Brener and Kluvitse, 1988). This paradigm required participants to state whether the external auditory stimulus was synchronous or asynchronous with their own heartbeat. Series of 10 auditory tones were presented, triggered by the participant's ECG R-wave and delivered with delays of 300, 400, 500, and 600 ms after the R peak. Each delay was repeated 14 times, pseudo-randomized across all trials (56 trials in total). Participants were instructed to focus simultaneously on both their own heartbeat and the external tone: “Please state whether the acoustic tone was synchronous or asynchronous with your heartbeat.”

Participants also completed an attention control task, in which participants were asked to detect a deviation in pitch from the standard feedback tone (Critchley et al., 2004). An oddball paradigm was employed in which 10 heartbeat tones were either presented all at the same frequency of 800 Hz (*n* = 28 trials), or one tone was randomly presented at the deviant pitch of 785 Hz (*n* = 28 trials). Participants received the following instructions: “Please state whether all tones in each trial series were of the same frequency or not.”

Upon completion of these tasks, participants were immediately asked to estimate the subjective task difficulty and performance accuracy on a five-point Likert scale (1–5): “How good do you think you were at [tone/heartbeat detection]?” and “How hard do you think [tone/heartbeat detection] was?”

### Psychometric assessments

#### Emotional disposition

Based on the observation that higher levels of interoceptive ability are associated with higher levels of trait anxiety and lower levels of depressive symptoms (Domschke et al., 2010; Stevens et al., 2011), all participants completed the widely used Trait-Anxiety-Inventory (STAI-T; Radloff, 1977; Spielberger, 1989) and the German version of the Center for Epidemiologic Studies Depression Scale (CESD; Radloff, 1977; German: Allgemeine Depressionsskala, ADS; Hautzinger et al., 1993). Internal consistency ranges between 0.86 and 0.95 (Cronbach's alpha) for the STAI-T and 0.89 and 0.92 for the ADS.

#### Musical performance anxiety (MPA)

To account for the specificity of MPA, musicians also completed the German version of Cox and Kenardy's Performance Anxiety Questionnaire (1993). The Bühnenangstfragebogen (BAF, Fehm et al., 2002) shows good internal consistency (Cronbach's alpha = 0.88) and contains 20 items to assess cognitive as well as bodily symptoms of performance anxiety (e.g., “I worry about my performance.” and “I feel tense in my stomach.”). The frequency of each symptom is indicated on a 5-point Likert scale with separate columns for solo and ensemble situations respectively. For simplicity, we will stick to the terms MPA solo and MPA group throughout this paper.

#### Extraversion

We selected this subscale from the German version of the NEO-Five Factor Inventory by Costa and McCrae (1992) and Borkenau and Ostendorf (1993) in addition to MPA, as recent studies indicated a negative correlation between MPA and extraversion (Thomas and Nettelbeck, 2014).

#### Body-centered awareness

We added two measures of body-centered awareness to test if interoceptive accuracy may be intrinsically related to a more prevalent (or dispositional) focus on bodily states.

The German version of the Self-Consciousness Scale (SCS; Fenigstein et al., 1975; German: Fragebogen zur Erfassung dispositionaler Selbstaufmerksamkeit, SAM; Filipp and Freudenberg, 1989), is a 23-item questionnaire that measures individual differences in public and private self-consciousness. Fenigstein et al. (1975) defined private self-consciousness as the tendency to introspect and examine one's inner self and feelings, whereas public self-consciousness referred to the awareness of the self as others may view it. Normative data for the German version indicated good internal reliability for both the public (α = 0.86) and the private (α = 0.87) self-consciousness subscales (Hinz et al., 2010).

Another form of body-centered awareness is represented in the attention to painful stimuli. This construct was assessed with the Pain Vigilance and Awareness Questionnaire (PVAQ; McCracken, 1997). The PVAQ consists of 16 items reflecting the individual's behavior over the last 2 weeks to indicate how frequently, on a six-point scale from zero (never) to five (always), each item is a true description of their behavior (e.g., “I seem to be more conscious of pain than others”). Normative data for the German version of the PVAQ (Roelofs et al., 2003) indicated good internal consistency for the entire questionnaire (α = 0.83) as well as for its subscales (attention of pain = 0.85, attention to changes in pain = 0.80).

### Statistical analyses

Data were analyzed with IBM SPSS statistics (IBM Corp. Released 2011. IBM SPSS Statistics for Mac, Version 20.0. Armonk, NY: IBM Corp.). Effect sizes for *t*-tests/ANOVA and regression analyses are reported as Cohen's *d* and *f*^2^, and interpreted as follows: *d* = 0.2 or *f* ^2^ = 0.02—small effect size; *d* = 0.5 or *f* ^2^ = 0.15—medium effect size; and *d* = 0.8 or *f* ^2^ = 0.35—large effect size (Cohen, 1988).

#### Group differences

##### Musical experience

We tested differences in musical experience between singers and string-players with *t*-tests for independent samples.

##### Interoceptive accuracy

To test the hypothesis that interoceptive accuracy differs between singers, string-players, and non-musicians, we first entered the four delays in heart-tone feedback (300, 400, 500, and 600 ms) into a 4 × 3 (delays × group) repeated measures ANOVA to assess a linear trend of percent synchronous ratings across all delays. However, because a simpler analysis yielded similar results (see Wiens and Palmer, 2001), we report this latter approach when presenting results. Accordingly, we restricted the analysis to the shortest (300 ms) and the longest (600 ms) heart-tone feedback delay based on previous observations that tones delivered around 200–300 ms after the R-wave are mostly perceived as synchronous, whereas tones delivered around 500–600 ms are mostly perceived as asynchronous (Brener et al., 1993; Wiens et al., 2000; Wiens and Palmer, 2001). Responses were coded as correct when heart-tone feedback delivered with 300 ms delay was either identified as synchronous or when heart-tone feedback delivered with 600 ms delay was identified as asynchronous. The percentage of correct answers represented the individual interoceptive accuracy, whereas the percentage of correctly identified deviant tones represented attention.

Differences in interoceptive accuracy between groups were assessed with a One-way ANOVA. Subsequently, planned comparisons (*t*-tests) were conducted to test the hypotheses that interoceptive accuracy was higher in musicians compared to non-musicians (singers vs. non-musicians, string-players vs. non-musicians) and higher in singers compared to string-players. The same procedure was repeated with the attention control condition.

##### Psychometric differences

Group differences in dispositional traits (STAI-T, SCS, CESD, PVQA, and extraversion) were analyzed using One-way ANOVAs. We reported Welch F-ratio when the assumption of homogeneity of variance was violated. Planned comparisons (*t*-tests) were conducted for the effects of musicianship (singers vs. non-musicians, string-players vs. non-musicians) and musician type (singers vs. string-players). If the sphericity assumption was violated, then degrees of freedom corrections were applied. Differences in solo and ensemble MPA between string-players and singers were assessed using an independent samples *t*-test.

Normative data were available for the STAI-T, SCS, CESD, PVQA, and the extraversion scale. In order to identify abnormal responses, group means were compared against the normal range in the general population.

##### Correlational analyses

Pearson's correlations were computed for each group separately to explore the relationship among psychometric variables as well as their correlation with interoceptive accuracy. Statistical significance was accepted at *p* < 0.05 using two-sided tests across all analyses.

#### Regression analyses

We computed hierarchical multiple regressions (HMR) to ascertain the extent to which accumulated training and dispositional traits can predict performance in the heartbeat-detection task. Selection of dispositional traits was based on correlation matrixes. That is, psychometric variables were only entered in the model when they were correlated with the accuracy of heartbeat discrimination and excluded when they were statistically unrelated (*p* > 0.10). In order to test the relative contribution of accumulated musical training to heartbeat-detection accuracy in singers and string players, we used multiple regressions implementing the Potthoff method (Potthoff, 1978; see also Weaver and Wuensch, 2013). This was followed by a regression model that tested the incremental value of the interaction between traits and training in addition to the main effect of accumulated training.

## Results

### Group differences

#### Musical training

Singers (M_age_ = 17.30, *SD* = 2.84) commenced significantly later with training [*t*_(23)_ = 12.23, *p* = 0.00; Cohen's *d* = 4.896] than string players (M_age_ = 6.75, *SD* = 0.97), who homogeneously commenced during early childhood. This difference also accounted for a significantly lower amount of accumulated training (M_hours_ = 6.440, *SD* = 3.674) in singers [*t*_(23)_ = −4.59, *p* = 0.00; *d* = −1.837] compared to string players (M_hours_ = 19.367, *SD* = 9.419).

#### Interoceptive accuracy

**Figure 1:**
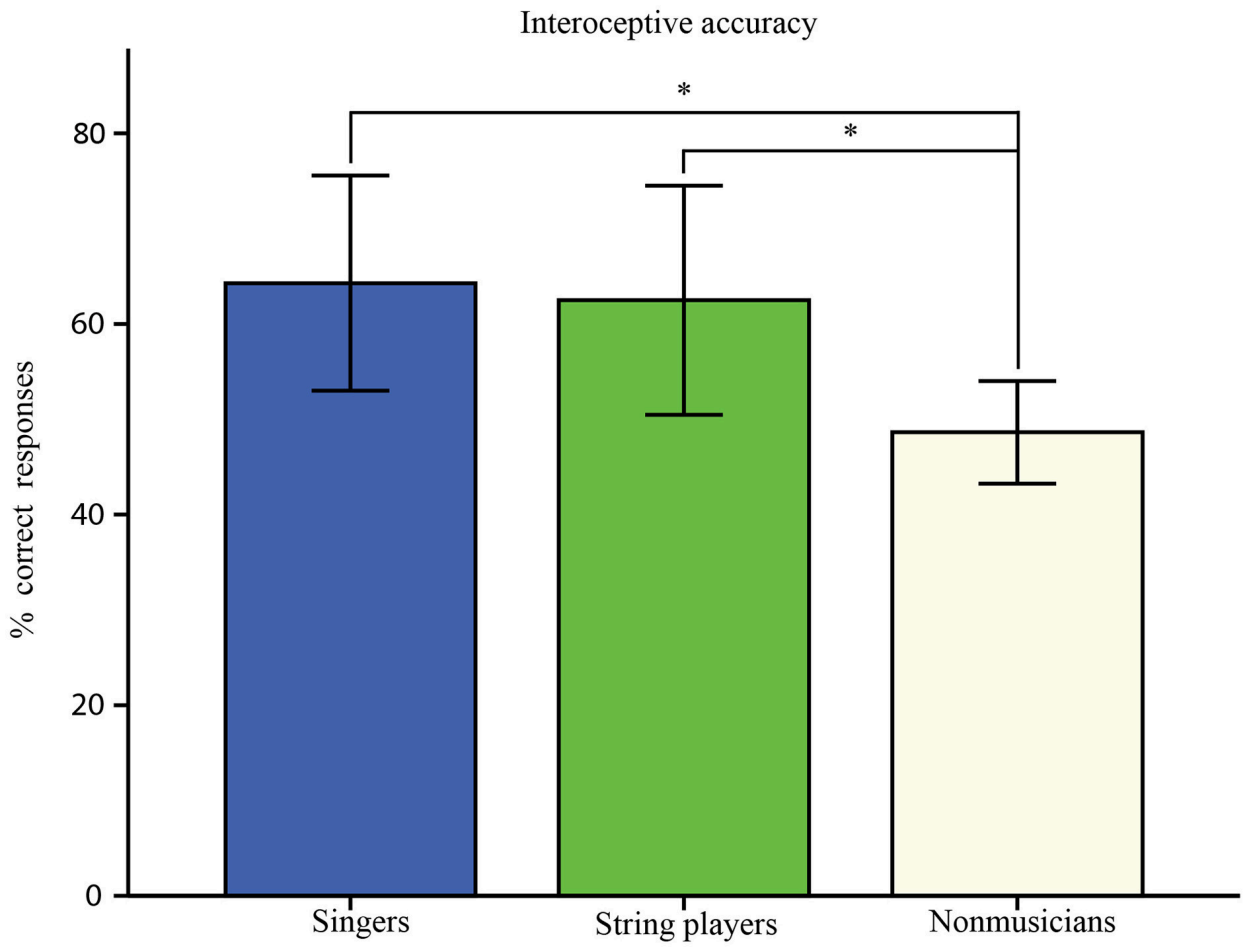
**Percent of correct heartbeat simultaneity judgments across 300 and 600 ms delays in R-peak auditory feedback**. Judgments were considered as correct when the former and the latter delays were identified as synchronous and asynchronous respectively (see also Brener et al., 1993; Wiens and Palmer, 2001). ^*^Indicates a significant difference in interoceptive accuracy between groups (*p* < 0.05).

Shapiro-Wilk tests indicated that interoceptive accuracy (i.e., the percentage of correct heartbeat-detection responses) was normally distributed across groups. Interoceptive accuracy varied between 39 and 92% correct responses in trained singers (*M* = 64, *SD* = 18.7), 39 and 100% in trained string players (*M* = 62.5, *SD* = 16.7), and 32 and 61% in non-musicians (*M* = 48.6, *SD* = 8.7, see also Table 1). There was a significant effect of group on interoceptive accuracy [*F*_(2, 20.02)_ = 5.27, *p* = 0.015; *d* = 2.74]. The assumption of homogeneity of variance was violated; therefore, the Welch F-ratio was reported. As shown in Figure 1, the mean interoceptive accuracy was higher in both singers [*t*_(17.22)_ = 2.73, *p* = 0.014; *d* = 1.07] and string-players compared to non-musicians [*t*_(15.39)_= 2.31, *p* = 0.035; *d* = 0.62], whereas no significant difference was detected between musicians.

#### Attention

Judgment accuracy in deviant tone detection (i.e., percentage correct responses) was equally high in singers (96.4–100%, *M* = 99.5, *SD* = 1.10), string-players (96.4–100%, *M* = 99.6, *SD* = 1.10), and non-musicians (94.6–100%, *M* = 99.0, *SD* = 1.00). A One-way ANOVA revealed no significant differences between groups.

#### Psychometric differences

One-way ANOVAs yielded no differences between groups with respect to dispositional traits. Moreover, all psychometric test results where within typical limits of normative data. In musicians, an independent samples *t*-test revealed that musical performance anxiety MPA was significantly lower for solo situations in singers (*M* = 46.3, *SD* = 9.89) than in string-players [*M* = 59.67, *SD* = 12.73; *t*_(23)_ = −2.94, *p* = 0.007; *d* = −1.177], whereas ensemble MPA did not achieve significance [*t*_(23)_ = −1.99, *p* = 0.058; *d* = −0.797].

A One-way ANOVA yielded an effect of group on self-reported interoceptive confidence [*F*_(2, 35)_ = 3.37, *p* = 0.46], which was due to significantly higher confidence in singers [singers: *M* = 3.0, *SD* = 0.91; *t*_(2, 35)_ = 2.48, *p* = 0.018, *d* = 2.86] compared non-musicians (*M* = 2.23, *SD* = 0.72). The difference between string-players (*M* = 2.83, *SD* = 0.72) and non-musicians, however, was not significant [*t*_(2, 35)_ = 1.9, *p* = 0.066]. No difference was detected between the two groups of musicians.

#### Correlation analyses

Singers (Table 1): Interoceptive accuracy was highly correlated with accumulated practice [*r* = 0.628, *p* = 0.002]. As expected, trait anxiety and depressive symptoms were positively correlated [*r* = 0.712, *p* = 0.006] and extraversion was negatively correlated with trait anxiety [*r* = −0.563, *p* = 0.045]. Moreover, solo MPA was negatively related with accumulated practice [*r* = −0.595, *p* = 0.032] but positively related with pain vigilance [*r* = 0.612, *p* = 0.026]. Interoceptive confidence was unrelated with interoceptive accuracy. Only heartbeat discrimination difficulty [*r* = −0.795, *p* = 0.001] and private self-attention [*r* = 0.586, *p* = 0.035] showed significant correlations with interoceptive confidence.

String-players (Table 2): Interoceptive accuracy was uncorrelated with accumulated practice. High correlations were observed between solo and ensemble musical performance anxiety [*r* = 0.793, *p* = 0.002]. Ensemble MPA correlated with depressive symptoms [*r* = 0.600, *p* = 0.039]. Solo MPA was also significantly related with trait anxiety [*r* = 0.592, *p* = 0.043]. Trait anxiety in turn was significantly related to pain vigilance [*r* = 0.601, *p* = 0.039], public self-awareness [*r* = 0.663, *p* = 0.019], and depressive symptoms [*r* = 0.611, *p* = 0.035]. Extraversion correlated positively with accumulated practice [*r* = 0.628, *p* = 0.029]. Interoceptive confidence was unrelated with interoceptive accuracy but significantly correlated with heartbeat discrimination difficulty [*r* = 0.600, *p* = 0.039].

**Table 2:** **Correlation matrix in trained singers**.

	**1**	**2**	**3**	**4**	**5**	**6**	**7**	**8**	**9**	**10**	**11**
1. Interoceptive accuracy	1										
2. Accumulated practice	0.628	1									
	0.022^*^										
3. Interoceptive confidence	0.402	−0.055	1								
	0.174	0.859									
4. Task difficulty	−0.370	−0.058	−0.795^**^	1							
	0.213	0.850	0.001								
5. PVAQ	−0.489	−0.363	−0.164	0.478	1						
	0.090	0.222	0.592	0.098							
6. SAM-Private	0.281	0.045	0.586^*^	−0.464	0.209	1					
	0.352	0.883	0.035	0.110	0.494						
7. SAM-Public	−0.287	−0.072	−0.127	0.259	0.411	−0.108	1				
	0.341	0.816	0.679	0.392	0.163	0.725					
8. ADS	0.315	0.539	−0.473	0.110	−0.240	−0.393	0.042	1			
	0.294	0.057	0.102	0.720	0.429	0.184	0.890				
9. STAI-T	−0.014	0.144	−0.465	0.226	0.090	−0.327	0.534	0.712^**^	1		
	0.963	0.639	0.109	0.457	0.771	0.276	0.060	0.006			
10. Extraversion	0.536	0.334	0.443	−0.355	−0.208	0.372	−0.308	−0.034	−0.563^*^	1	
	0.059	0.264	0.129	0.235	0.496	0.211	0.306	0.913	0.045		
11. MPA Solo	−0.268	−0.595^*^	0.175	0.017	0.612^*^	0.080	0.410	−0.170	0.347	−0.461	1
	0.377	0.032	0.566	0.956	0.026	0.794	0.165	0.578	0.246	0.113	
12. MPA Ensemble	−0.106	−0.540	0.330	−0.202	0.144	0.204	0.140	−0.364	−0.015	−0.159	0.485
	0.731	0.057	0.271	0.508	0.638	0.503	0.648	0.221	0.960	0.603	0.093

** *Correlation is significant at the 0.01 level (2-tailed)*.

* *Correlation is significant at the 0.05 level (2-tailed)*. *Group, Singers*.

Non-musicians (Table 3): Pain vigilance correlated with interoceptive confidence [*r* = 0. 636, *p* = 0.020] and public self-attention [*r* = 0. 554, *p* = 0.050]. No other significant correlations were observed.

**Table 3:** **Correlation matrix in string-players**.

	**1**	**2**	**3**	**4**	**5**	**6**	**7**	**8**	**9**	**10**	**11**
1. Interoceptive accuracy	1										
2. Accumulated practice	0.061	1									
	0.851										
3. Interoceptive confidence	0.287	−0.158	1								
	0.366	0.624									
4. Task difficulty	−0.314	0.250	−0.600	1							
	0.321	0.432	0.039								
5. PVAQ	0.222	0.437	0.201	0.008	1						
	0.489	0.155	0.532	0.980							
6. SAM-Private	−0.007	0.192	−0.050	0.049	−0.002	1					
	0.983	0.549	0.876	0.880	0.996						
7. SAM-Public	0.467	0.368	−0.007	0.333	0.570	0.457	1				
	0.126	0.240	0.984	0.290	0.053	0.135					
8. ADS	0.414	0.278	0.354	−0.224	0.368	0.238	0.374	1			
	0.181	0.382	0.259	0.483	0.239	0.456	0.231				
9. STAI-T	0.365	0.175	0.256	0.133	0.601^*^	−0.004	0.663^*^	0.611^*^	1		
	0.244	0.588	0.421	0.679	0.039	0.990	0.019	0.035			
10. Extraversion	−0.026	0.649^*^	−0.064	0.449	0.471	−0.087	0.326	−0.142	0.042	1	
	0.936	0.022	0.844	0.143	0.122	0.788	0.301	0.660	0.896		
11. MPA Solo	0.004	0.004	0.013	−0.155	0.121	−0.326	−0.013	0.483	0.592^*^	−0.368	1
	0.990	0.990	0.967	0.631	0.709	0.301	0.968	0.112	0.043	0.239	
12. MPA Ensemble	−0.030	0.114	0.335	−0.447	0.169	−0.091	−0.182	0.628^*^	0.455	−0.323	0.793^**^
	0.927	0.724	0.287	0.146	0.600	0.778	0.572	0.029	0.137	0.306	0.002

** *Correlation is significant at the 0.01 level (2-tailed)*.

* *Correlation is significant at the 0.05 level (2-tailed)*. *Group, String-players*.

#### Regression analyses

Multiple linear regression analysis was used to develop a model for predicting interoceptive accuracy from dispositional traits and accumulated musical training. Transformed data (z-score) revealed one extreme case (100% accuracy) that scored two standard deviations above the group average in string players, which was removed prior to regression analysis. Trait and self-report variables were selected when their correlation with interoceptive accuracy was *p* < 0.1. Correlation statistics are shown in Tables 2–4. Based on this criterion, a multiple linear regression was performed in singers to predict interoceptive accuracy based on extraversion (IV^1^) and pain vigilance (IV^2^). The multiple regression model with two psychometric predictors in the first block just failed to reach significance at *R*^2^ = 0.463, *F*_(2, 10)_ = 3.87, *p* = 0.057, Cohen's *f* ^2^ = 0.77. As can be seen in Table 5, neither the extraversion scale with positive nor the pain vigilance with negative regression weights was sufficient to predict interoceptive accuracy.

**Table 4:** **Correlation matrix in non-musicians**.

	**1**	**2**	**3**	**4**	**5**	**6**	**7**	**8**
1. Interoceptive accuracy	1							
2. Interoceptive confidence	−0.315	1						
	0.295							
3. Task difficulty	−0.037	−0.497	1					
	0.904	0.084						
4. PVAQ	−0.075	0.636^*^	−0.141	1				
	0.808	0.020	0.645					
5. SAM-Private	0.254	−0.202	0.032	0.366	1			
	0.402	0.509	0.918	0.219				
6. SAM-Public	0.261	−0.009	0.202	0.554^*^	0.310	1		
	0.388	0.977	0.507	0.050	0.303			
7. ADS	0.386	0.250	−0.101	0.035	−0.186	0.136	1	
	0.193	0.410	0.742	0.909	0.544	0.659		
8. STAI-T	0.426	0.134	−0.247	0.187	0.091	0.414	0.538	1
	0.146	0.662	0.417	0.540	0.767	0.159	0.058	
9. Extraversion	0.194	−0.398	0.228	−0.099	0.401	0.178	−0.170	−0.267
	0.525	0.179	0.454	0.748	0.174	0.560	0.579	0.378

* *Correlation is significant at the 0.05 level (2-tailed)*. *Group, Non-musicians*.

**Table 5:** **Multiple regression with dispositional traits as IV**.

**Model**		**b**	***SE* b**	***β***
Step 1	Constant	59.212	24.133	
Extraversion		1.054	0.564	0.454
Pain Vigilance (PVAQ)		−0.663	0.408	−0.395

*The dependent variable was interoceptive accuracy. R^2^ = 0.436. $R_{adj.}^{\text{2}}$= 0.323. p = 0.057, f^2^ = 0.77*.

A multiple regression was performed to predict interoceptive accuracy from accumulated musical training. We implemented the Potthoff method (1978; Weaver and Wuensch, 2013) to test the difference between regression coefficients in singers relative to string players. The model included the continuous predictor (accumulated musical training), the grouping variable (singers = 1, strings = 0), and the interaction term (training^*^group). This model accounted for 39.1% of the variance in interoceptive accuracy [*R*^2^ = 0.391, *F*_(3, 20)_ = 4.28, *p* = 0.017, *f* ^2^ = 0.64]. As can be seen in Table 6, of the individual terms in the model only the interaction (training^*^group) became significant. This indicates that the regression slope of singers (i.e., predicting accuracy from training) was significantly steeper than in string players.

**Table 6:** **Multiple regression with accumulated training and group as IV in musicians**.

**Model**		**b**	***SE* b**	***β***
Step 1	Constant	44.864474	10.794510	
Acc. musical training		0.000701	0.000488	0.397510
Group (singers = 1, strings = 0)		−3.506074	13.601632	−0.104154
Training^*^Group		0.002859	0.001228	0.703673

The dependent variable was interoceptive accuracy. The Potthoff method (1978) was employed to test the difference in regression slopes between singers and string players. R^2^ = 0.391, $R_{adj.}^{\text{2}}$ = 0.299; p = 0.017

Given this significant difference in regression slopes between groups, we set up an additional regression model only for the singers (Table 7). In the first step, the main effect of accumulated musical practice proofed to be a highly significant predictor of interoceptive accuracy, accounting for 44.4% of the variance [*R*^2^ = 0.490, *F*_(1, 11)_ = 10.60, *p* = 0.008, *f* ^2^ = 0.96]. In the second step, we additionally modeled the interaction terms between musical training with extraversion and pain vigilance respectively. The latter model was also significant [*R*^2^ = 0.624, *F*_(3, 9)_ = 4.98, *p* = 0.026, *f* ^2^ = 0.166]. However, the amount of additionally explained variance (13.4%) remained non-significant compared to the first model [$R_{\text{change}}^{2}$ = 13.4, *F*_(3, 9)_ = 1.60, *p* = 0.254].

**Table 7:** Multiple regression with accumulated training and trait^*^training as IV in singers.

**Model**		**b**	***SE* b**	***β***
Step 1	Constant	41.358399	8.032249	
Acc. musical training		0.003560	0.001094	0.700444
Step 2	Constant	50.784633	9.557426	
Acc. musical training		0.001875	0.002862	0.368905
Extraversion^*^Training		0.000095	0.000079	0.759663
Pain Vigilance^*^Training		−0.000076	0.000044	−0.544230

*The dependent variable was interoceptive accuracy. Step 1: R^2^ = 0.490621, $R_{adj.}^{\text{2}}$= 0.444314; p = 0.007667. Step 2: R^2^ = 0.624385, $R_{change}^{\text{2}}$= 0.133764, p_(Fchange)_ = 0.253898*.

## Discussion

**Figure 2:**
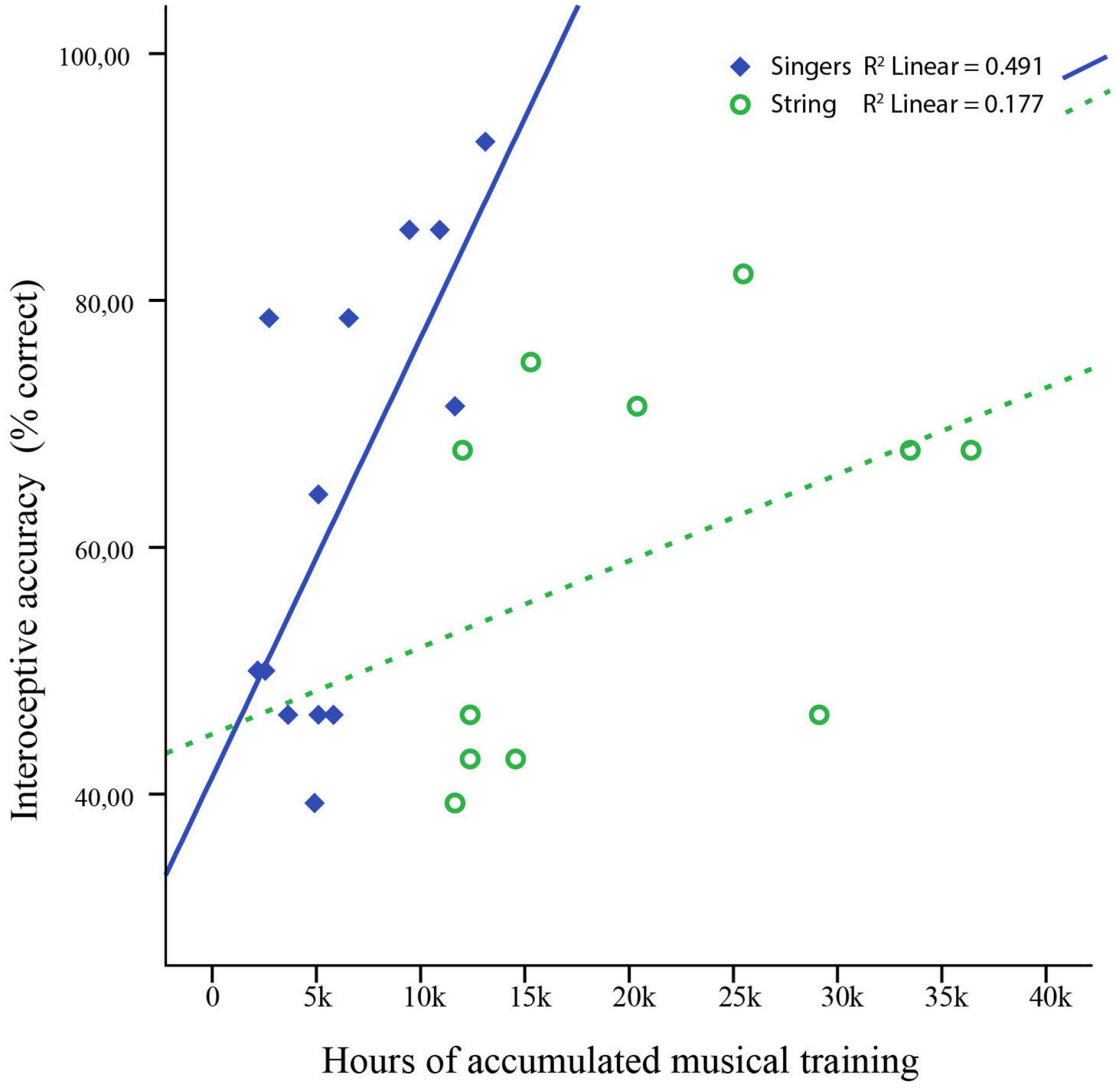
**Results from regression analyses, testing the correlation between heartbeat detection accuracy (simultaneity judgments) and the total amount of accumulated musical training in singers and string players respectively**.

We measured interoceptive accuracy in non-musicians and two groups of professional musicians (i.e., trained singers of classical music and professional string players) using a well-established heartbeat discrimination paradigm (Critchley et al., 2004). Across musicians, we found higher interoceptive accuracy compared to non-musicians, whereas no significant differences were found between singers and string-players (Figure 1). Despite similar performance in heartbeat discrimination, accumulated musical practice predicted interoceptive accuracy only in singers (about 49% of variance explained) but not in string players (Table 6 and Figure 2). The influence of dispositional traits furthermore yielded no significant main effect in any of the groups (see also Table 5). Extraversion and pain vigilance interacting with training respectively explained additional variance in interoceptive accuracy in singers (Table 7), yet this difference was non-significant compared to the effect of training alone. Together, these data provide first evidence that interoceptive accuracy may be enhanced in musicians. Results in singers furthermore imply that musical training accounts for this effect, independent from differences in dispositional traits.

### Interoceptive accuracy and musicianship

The ability to perceive signals that arise from within your body is considered to be a stable trait and remains unchanged even after the explicit training of internally focused attention (Khalsa et al., 2008; Ainley et al., 2013). Heartbeat discrimination, in which individuals are asked to compare externally generated acoustic signals to the rhythm of their own heartbeat, is a way to measure interoceptive accuracy but has proven to be a difficult task. Healthy participants often perform only around chance level, depending on the amount of delay added the external cue (Wiens and Palmer, 2001). This floor effect can prevent the detection of differences between groups based on their dispositional characteristics, for which heartbeat tracking tasks are thought to be more sensitive (see Domschke et al., 2010). The increased difficulty of heartbeat discrimination paradigms, however, has been partially explained by Pennebaker's “competition of cues” hypothesis (Pennebaker, 1982), who argued that attention to external environmental stimuli lowers an individual's capacity to detect internally generated physical sensations due to a competition of exteroceptive and interoceptive signals. The competition of cues hypothesis could therefore explain the poor performance in non-musicians when we also consider the salience of external auditory cues, which may arguably be higher in musicians. Indeed, interoceptive accuracy in non-musicians with below-median performance has shown to increase when they integrated the visual information of self-photographs as opposed to photos of strangers (Maister and Tsakiris, 2014). Likewise, interoceptive accuracy can also be enhanced during mirror self-observation (Weisz et al., 1988; Ainley et al., 2012) and attention to self-relevant external cues (Ainley et al., 2013). It follows that the integration of self-relevant external (or multimodal) information may lead to a facilitation of interoceptive accuracy if concordant with the task requirements and the context (Suzuki et al., 2013). Conceivably, musicians who are trained in multimodal integration might therefore also show heightened sensitivity to interoceptive cues when simultaneously processing related acoustic information. This concept could be validated with a comparable group of experts, such as professional dancers. Although we measured only heartbeat discrimination, we speculate that musicians would have also scored at least equal or higher in a heartbeat-tracking task, as both tasks are highly correlated (Garfinkel et al., 2015).

Extensive musical training aims at performing fine-motor sequences at high metric accuracy, which crucially requires an optimized interplay between multisensory and motor systems in both singers and instrumentalists. In fact, repetitive and simultaneous integration of multisensory, motor, and cognitive information is a defining characteristic of professional musicians (Zatorre et al., 2007; Schlaug, 2015). Consequently, task-related structural and functional adaptive changes in the brain have been observed both within (Elbert et al., 1995; Lotze et al., 2003; Altenmüller, 2008; Kleber et al., 2010) and between sensorimotor areas (Gaser and Schlaug, 2003; Hirata et al., 2004; Barnes-Burroughs et al., 2005; Baumgartner et al., 2006; Pantev et al., 2009; Herholz and Zatorre, 2012). In the same vain, sensory re-weighting of afferent signals and related distortions of mental body representations have been reported in the presence of chronic pain (Tsay et al., 2015), whereas increased pain sensitivity has also been observed in healthy musicians (Zamorano et al., 2014).

In this sense, our results suggest that enhanced interoceptive accuracy in both groups of musicians could be a consequence of long-term musical training. Although both groups performed equally in the accuracy task (Figure 1), accumulated training predicted interoceptive accuracy only in singers but not in string-players (see Table 6 and Figure 2). Foster and Zatorre (2010) reported brain areas that were anatomically related to both behavioral performance and accumulated training, yet they also found that this relationship still remained after removing the effect of training. In our study, accumulated training explained about 49% of variance in interoceptive accuracy in singers, which allows for the possibility that pre-existing tendencies might have played a role too. Moreover, singing has been linked to greater phylogenetic continuity, as singers can use the same vocal gestures to project feelings and discrete emotions in music performance as in social interactions (Juslin and Laukka, 2003), which might involve the interoceptive system to a greater extent. The differences in predicting interoception from training between musicians, however, may also be related to differences in sensitive periods. It has been demonstrated in instrumentalists that early training (<7 years) enhanced both performance and brain plasticity compared those musicians who were trained later (>7 years), thus highlighting the interaction between training and maturation (Bailey and Penhune, 2013; Bailey et al., 2014). In singers, maturation differs from instrumentalists due to the protracted time course of speech motor development (Smith and Zelaznik, 2004; Smith, 2006). Correspondingly, most singers also commence considerably later with formal training (Ericsson et al., 1993; Jørgensen, 2002; Ericsson, 2008; Kleber et al., 2010). In our study, string players commenced significantly earlier (6.8 vs. 17 years) and accumulated more training than singers (~19,000 vs. ~6000 h). Therefore, effects of accumulated training on interceptive accuracy could have been veiled by a ceiling effect in adult string players, corresponding to the observation that interoceptive accuracy didn't seem to increase more after about 10,000 h of training (Figure 2).

Support for training related effects comes from the neurosciences. Yu et al. (2010) demonstrated that repeated multisensory exposure enhances the responsiveness of neurons to multisensory inputs, which in turn shapes their ability to integrate multisensory information. This effect was observed across the life span and may be applied to other stimulus conditions once the multisensory capability is learned. At the macro anatomical level, the anterior insula represents the key structure that has been associated with multisensory integration and interoceptive awareness (Ádám and Pennebaker, 1998; Craig, 2009). Individual differences in interoception have been successfully attributed to the degree of expansion of right anterior and adjacent orbitofrontal cortices (Craig, 2004). Critchley et al. (2004) showed that activation of the right anterior insula (AI) can be positively related to interoceptive accuracy, negative emotional experience, and trait anxiety, whereas interoceptive accuracy also predicted gray matter volume. In contrast, destruction of the right AI diminished heartbeat awareness (Ursino et al., 2014).

In a previous fMRI study with trained singers, we found that activity of the right AI distinguished between singers and non-singers during singing with and without anesthetized vocal fold mucosa (Kleber et al., 2013). The insula has also been implicated in the processing of musical tempo, melody, and emotion (Platel et al., 1997; Koelsch, 2005; Craig, 2009; Thaut et al., 2014), where it supposedly contributes to the process of predictive coding (Seth et al., 2011; Seth, 2013; Seth and Critchley, 2013; Ainley et al., 2014). Prediction refers to “any type of processing, which incorporates or generates not just information about the past and the present, but also future states of the body and of the environment” (Bubic et al., 2010, p. 1). Perception, on the other hand, entails knowledge about the origins of sensations based on a-priori acquired experience, which enables the brain to generate predictive models for the estimation of action parameters that are required to achieve the desired outcome. The ability to perceive and integrate salient (intero- and exteroceptive) sensory information therefore plays an indispensable role in sensorimotor control, emotions, and cognition (Vuust et al., 2009; Seth et al., 2011; Seth, 2013; Seth and Critchley, 2013; Cauda et al., 2014). Any mismatch between feedback and prediction would compute an error signal to update the model (Adams et al., 2013), while accurate predictions solve problems inherent to sensorimotor control, such as the time lag of incoming sensory feedback (Franklin and Wolpert, 2011; Hickok, 2012). Superior predictive motor control has been demonstrated in musicians, who show smaller compensatory responses to unexpected shifts in real-time pitch feedback (Jones and Keough, 2008; Zarate and Zatorre, 2008). Considering both the association between the insula with interoception and multisensory integration on one hand and the experience-dependent role of the insula in motor control on the other hand, the insula could be major factor in explaining the results of enhanced interoceptive accuracy in musicians.

### Psychological interactions

Despite the prevalently described relationship between cardiac interoceptive accuracy and dispositional traits (for review, see Domschke et al., 2010), we did not find strong evidence for such a connection in our study. Dispositional traits were all within normal limits and equally distributed across groups. Moreover, none of the psychometric variables significantly predicted interoceptive accuracy in any of the groups. In trained singers, the main effects of extraversion and pain vigilance just failed to predict heartbeat detection performance from these variables. Adding the interactions of extraversion and pain vigilance respectively with accumulated training, another 13% of variation in interoceptive accuracy was explained in addition to training alone. Although this difference was not statistically significant, dispositional traits might at least partly contribute to interoception. Perhaps dispositional traits that affect interoception could also affect the choice of becoming a musician, yet little is known about the relationship between personality and musical involvement (Corrigall et al., 2013). Therefore, it seems more likely that opportunity, early experiences, and parental support play a bigger role (Howe et al., 1998; McPherson and Davidson, 2002; McPherson and Williamon, 2006).

The lack of main effects on interoceptive accuracy is interesting considering the vast interrelations between psychometric variables in musicians compared to non-musicians (Tables 2–4). We speculate that these differences are mainly related to the specific conditions that accompany professional musical activity, which can lead to the experience of musical performance anxiety in about 25–56% of musicians (Kenny et al., 2004; Nieuwenhuys and Oudejans, 2012; Papageorgi et al., 2013). Moreover, performing music is a strong stimulus that can trigger more intense emotion-related psychophysiological responses than musical perception alone (Nakahara et al., 2011). Although string players seem to experience more negative emotions than singers, we found no evidence for an influence on interoceptive accuracy. The negative correlation between extraversion and trait anxiety in singers possibly reflects their ability to communicate and act on stage, which might favor the personality trait of extraversion (Corrigall et al., 2013; Cameron et al., 2015).

### Limitations

For the general interpretation of these data, several limitations have to be taken into account. Sample size was considerably lower compared to other studies investigating interoception in healthy normal populations. The lower sample size was mainly due to our choice to select highly homogeneous samples of trained professional classical singers and string players in order to validate the assumed incremental effects of training specificity on interoceptive accuracy. Therefore, we cannot exclude the possibility that other factors such as dispositional traits would affect interoception in a larger sample. For future studies, we recommend to include more musicians of various training modalities (e.g., wind, brass, string, and keyboard). Despite the sample size, however, effect sizes were large throughout analyses, indicating that our results represent meaningful differences in interoceptive accuracy between trained musicians and non-musicians.

## Conclusions

In conclusion, we presented first evidence indicating that interoceptive awareness can be enhanced in professional musicians compared to non-musicians, whereas dispositional traits were unrelated in all groups. In contrast to our initial hypothesis, we found no differences in interoceptive accuracy between singers and string-players. Our data in singers, however, suggest that musical training accounted for enhanced interoceptive accuracy. It is worth noting that non-musicians in our experiment scored on average lower than participants in other studies using the identical paradigm (Garfinkel et al., 2015). Given their large inter-individual variation of interoceptive accuracy, it is intriguing to speculate that musical training could have unknowingly driven higher scores in these groups.

### Conflict of interest statement

The authors declare that the research was conducted in the absence of any commercial or financial relationships that could be construed as a potential conflict of interest.
